# Influence of the Phase Composition of Titanium Alloys on Cell Adhesion and Surface Colonization

**DOI:** 10.3390/ma16227130

**Published:** 2023-11-11

**Authors:** Boris B. Straumal, Natalia Yu. Anisimova, Mikhail V. Kiselevskiy, Keryam M. Novruzov, Anna Korneva, Alena S. Gornakova, Askar R. Kilmametov, Silvana Sommadossi, Gregory Davdian

**Affiliations:** 1 Osipyan Institute of Solid State Physics, Russian Academy of Sciences, Ac. Osipyan Str. 2, Chernogolovka 142432, Russia; alenahas@issp.ac.ru (A.S.G.); davdian@issp.ac.ru (G.D.); 2 N.N. Blokhin National Medical Research Center of Oncology of the Ministry of Health of the Russian Federation (N.N. Blokhin NMRCO), Moscow 115478, Russia; n_anisimova@list.ru (N.Y.A.); kisele@inbox.ru (M.V.K.); nkeryam@gmail.com (K.M.N.); 3 Department of Casting Technologies and Artistic Processing of Materials, National University of Science and Technology “MISIS”, Moscow 119049, Russia; 4 Institute of Metallurgy and Materials Science, Polish Academy of Sciences, Reymonta Str. 25, 30-059 Cracow, Poland; a.korniewa@imim.pl; 5 Process and Material Sciences Laboratory, LSPM—CNRS, Bâtiments L1/L2, 99 Av. Jean-Baptiste Clément, 93430 Villetaneuse, France; askar.kilmametov@lspm.cnrs.fr; 6 Institute for Research in Engineering Sciences and Technologies National Council for Scientific and Technical Research, National University of Comahue, Buenos Aires 1400 (Q8300IBX), Patagonia, Neuquén 8300, Argentina; silvana.sommadossi@fain.uncoma.edu.ar

**Keywords:** titanium alloys, phases, microstructure, grain size, cell adhesion, surface colonization, hemolysis, cytotoxicity

## Abstract

The pivotal role of metal implants within the host’s body following reconstructive surgery hinges primarily on the initial phase of the process: the adhesion of host cells to the implant’s surface and the subsequent colonization by these cells. Notably, titanium alloys represent a significant class of materials used for crafting metal implants. This study, however, marks the first investigation into how the phase composition of titanium alloys, encompassing the volume fractions of the α, β, and ω phases, influences cell adhesion to the implant’s surface. Moreover, the research delves into the examination of induced hemolysis and cytotoxicity. To manipulate the phase composition of titanium alloys, various parameters were altered, including the chemical composition of titanium alloys with iron and niobium, annealing temperature, and high-pressure torsion parameters. By systematically adjusting these experimental parameters, we were able to discern the distinct impact of phase composition. As a result, the study unveiled that the colonization of the surfaces of the examined Ti–Nb and Ti–Fe alloys by human multipotent mesenchymal stromal cells exhibits an upward trend with the increasing proportion of the ω phase, concurrently accompanied by a decrease in the α and β phases. These findings signify a new avenue for advancing Ti-based alloys for both permanent implants and temporary fixtures, capitalizing on the ability to regulate the volume fractions of the α, β, and ω phases. Furthermore, the promising characteristics of the ω phase suggest the potential emergence of a third generation of biocompatible Ti alloys, the ω-based materials, following the first-generation α-Ti alloys and second-generation β alloys.

## 1. Introduction

Biomaterials have been recognized for their utility since antiquity, finding application in the restoration of various parts of the human body. However, their extensive adoption within the realm of medicine took a significant leap forward following the Second World War [1]. These materials now hold a pivotal role in enhancing the quality of life and increasing the life expectancy of aging populations worldwide. Presently, they serve as the building blocks for a wide array of medical devices, including vascular stents, artificial heart valves, endoprostheses for bones and joints (such as the shoulder, knee, hip, and elbow), ear reconstruction, facial surgery, and dental implants [2]. Notably, endoprostheses for the replacement of vertebrae, knee, and hip joints have gained exceptional popularity. These artificial biomaterials have revolutionized the treatment of conditions like osteoporosis, osteoarthritis, and various injuries. Diverse classes of materials, including metals, ceramics, and organic polymers, among others, are harnessed for these biological purposes [3,4,5,6]. Furthermore, a critical patient group comprises those affected by bone cancer, arising from primary tumors or metastases, necessitating the surgical replacement of skeletal components for health restoration. Irrespective of the medical context, achieving optimal contact between permanent implants and the bone is paramount. However, biomaterials also serve as temporary fixtures, for instance, in facial surgery, where removal becomes necessary after the patient’s bones have fully fused. In such instances, the fixtures must not integrate with the patient’s bones. Hence, a wide spectrum of requirements governs the formation of the interface between artificial biomaterials and human or animal tissues.

The selection of materials for medical implants and their design are intricately linked to the particular medical context and the unique characteristics of each patient. To ensure the optimal performance of an implant while minimizing the risk of rejection [7,8,9], several pivotal criteria must be carefully considered. These essential factors encompass precise mechanical properties [10,11], compatibility with the biological environment [12,13], robust resistance to corrosion and wear [5,14,15], and the absence of allergic reactions [16].

A diverse array of materials finds extensive application in the fabrication of surgical implants, encompassing metallic alloys, such as chromium–nickel stainless steel (e.g., 316LSS or 1X18H10T) [17], cobalt–chromium and nickel–chromium alloys [18,19], magnesium-based biodegradable alloys [4,13,20,21], biodegradable zinc alloys [22], titanium and its alloys [23,24,25,26,27,28,29,30,31], and multiprincipal alloys devoid of a predominant component [32,33,34]. Each of these materials serves specific niches within the field of medical practice, but the development of each presents unique challenges. One pressing concern is the gradual release of certain elements, such as nickel, cobalt, and chromium, due to slow corrosion within the body, potentially leading to toxic effects [35]. For instance, nickel is linked to contact dermatitis, cardiovascular disease, asthma, lung fibrosis, and respiratory tract cancer, while cobalt may impact the heart, thyroid, liver, and kidneys. Similarly, chromium has the potential to cause kidney and liver damage, pulmonary congestion, and edema. Another prevalent challenge revolves around fatigue failure, particularly noticeable in hip prostheses subject to repetitive loading and unloading over many years of use [36].

At present, titanium-based alloys stand as the top-choice materials for medical implants in clinical applications. This preference is underscored by the remarkable attributes exhibited by titanium and its alloys, including outstanding strength, low density (resulting in high specific strength), exceptional corrosion resistance, biocompatibility, a low elastic modulus, and a remarkable ability to seamlessly integrate with the body’s bones and tissues [1,2,3,5,10,11,15,23,24,25,26,27,28,29,30,31,37,38]. These qualities collectively make titanium and its alloys the gold standard for medical implant materials.

The exploration of using titanium for crafting prosthetic bones in animals dates back to the late 1930s. During this period, it became evident that titanium exhibited remarkable suitability for replacing femur bones in cats, a role that had previously been fulfilled by materials such as stainless steel or vitallium alloy, composed of cobalt, chromium, and molybdenum. The first-generation medical alloys of titanium predominantly consisted of the α-Ti phase. In contemporary times, high-purity titanium and VT6 alloy, recognized as Ti–6Al–4V ELI or Ti64, continue to reign as the predominant choices for manufacturing medical implants. Titanium alloys find extensive application across a spectrum of medical scenarios, ranging from dental implants and endoprostheses for facial surgery to the replacement of hip, knee, shoulder, and elbow joints. They also serve as essential components for bone-fixation devices like pins, screws, and plates, as well as housing materials for heart-rate motors, artificial heart valves, surgical instruments, and high-speed centrifuge components dedicated to the separation of blood constituents [39,40,41,42,43,44,45,46].

The mechanical properties of a material, along with its ability to withstand wear and corrosion, are intricately linked to its microstructure. In this context, titanium alloys emerge as a subject of particular significance due to their versatility. Through the manipulation of alloy composition and the application of high-temperature or high-pressure processing, a diverse array of microstructures can be achieved. Titanium exhibits three distinct modifications: low-temperature α-Ti, high-temperature β-Ti, and high-pressure ω-Ti [47,48]. Notably, the formation of the ω phase becomes notably pronounced when subjected to elevated levels of deformation and pressure [49,50,51,52,53].

Considerable research efforts have been dedicated to exploring various titanium alloys, incorporating different alloying elements. Theoretical investigations have ascertained that elements like neodymium, zirconium, molybdenum, and tantalum serve as suitable alloying agents capable of reducing the elastic modulus of β-titanium while preserving the alloy’s strength [11,54]. Consequently, the second generation of medical Ti-based alloys predominantly consisted of the β phase. Research findings have also revealed that introducing these β stabilizers in small quantities to titanium effectively lowers its elastic modulus. However, when the concentration of these alloying elements is increased, the elastic modulus starts to rise due to the emergence of the ω phase and the release of α-phase particles during material aging [55,56]. A critical aspect of these elements lies in their low toxicity, rendering them appealing for use in implant applications [57]. Building on this knowledge, metallurgists have developed several biomedical titanium alloys, featuring combinations of titanium, niobium, tantalum, and zirconium. Notably, some of these alloys, such as Ti–29Nb–13Ta–4.6Zr and Ti–35Nb–7Zr–5Ta, have undergone comprehensive scrutiny [58,59,60].

As highlighted earlier, the interaction between artificial biomaterials and human or animal tissues involves a wide range of stringent requirements. Unfortunately, a significant aspect of titanium alloys as implant materials has received relatively little attention from researchers. The long-term success of metal implants within the host’s body after reconstructive surgery hinges largely on the initial phase of this process, specifically the adhesion of host cells to the implant’s surface and the subsequent colonization of that surface by cells. It stands to reason that the adhesion and colonization dynamics may vary across the α, β, and ω phases of titanium alloys, assuming other material parameters remain constant or comparable. This study is dedicated to empirically investigating this aspect. The selection of materials for this research leverages the authors’ extensive experience in exploring α–β–ω phase transformations within titanium alloys [50,51,52,53]. The primary aim is to disentangle the direct influence of the proportion of the α, β, and ω phases while keeping other factors, such as composition or grain size, constant. The outcomes of this investigation, shedding light on the distinct roles of various Ti phases in biocompatibility, have the potential to empower materials scientists, engineers, and medical practitioners to enhance the development of Ti-based alloys tailored to medical applications. Furthermore, it may pave the way for the emergence of the third generation of biocompatible Ti alloys, notably the ω-based variants.

## 2. Experimental

### 2.1. Alloys Preparation and Structure Analysis

The study encompassed four binary titanium–iron alloys, comprising 0.5 ± 0.05 wt.% Fe, 1.0 ± 0.07 wt.% Fe, and 4.0 ± 0.21 wt.% Fe, alongside five binary titanium–niobium alloys, featuring 5.0 ± 0.05 wt.% Nb, 6.0 ± 0.05 wt.% Nb, 10.0 ± 0.1 wt.% Nb, 20.0 ± 0.1 wt.% Nb, and 30.0 ± 0.05 wt.% Nb. These alloys were crafted using pure titanium (99.98%, TI-1 grade, Special Metallrgy Ltd., Moscow, Russia), iron (99.97%, Special Metallrgy Ltd., Moscow, Russia), and niobium (99.97%, Special Metallrgy Ltd., Moscow, Russia) through a unique levitation method conducted within a pure argon atmosphere. In this innovative method, molten metal levitates within a “cold crucible”, comprised of a circular assembly of vertically oriented, water-cooled copper tubes. Surrounding the cold crucible, a water-cooled copper induction coil generates a potent magnetic field at supersonic frequencies, inducing Foucault eddy currents within the molten metal. This leads to intense heating, raising the metal to its melting point. Simultaneously, these eddy currents create a countering magnetic field around the molten material, which interacts with the primary magnetic field, producing Lorentz forces. These forces enable the liquid metal bath to levitate within a vacuum or inert atmosphere without making contact with the walls of the cold crucible.

The process began by cutting 0.7 mm thick discs with a 10 mm diameter from cylindrical ingots obtained earlier. Each sample was then securely sealed in a quartz ampoule and subjected to annealing within a vacuum environment at a residual pressure of 4 × 10^−4^ Pa. The annealing process involved a range of temperatures, including 1000, 950, 900, 800, 600, 570, and 470 °C, situated in distinct regions of the phase diagrams of titanium with iron and niobium (refer to Table 1). The heat treatment was meticulously executed to yield samples (a) predominantly featuring the β phase, (b) a blend of α and β phases, (c) exclusively the α phase, or (d) a combination of the α phase and the intermetallic compound. Consequently, a series of α + β samples was generated, each exhibiting varying proportions of the α and β phases, stemming from both (a) identical compositions or at (b) differing alloy-component concentrations. Following annealing, the samples underwent quenching in water, still within their respective ampoules. High-pressure torsion (HPT) was conducted utilizing a custom-built, computer-controlled apparatus crafted by W. Klement GmbH (Lang, Austria). This HPT procedure occurred at room temperature, employing 7 GPa of pressure, a deformation rate of 1 rpm, and five revolutions of the plunger. Initially, the sample thickness ranged from 0.6 to 0.7 mm, which was subsequently reduced to 0.35 mm after HPT treatment. The design of the HPT procedure aimed to create samples featuring (a) ultrafine grains with similar chemical and phase compositions, as well as (b) combinations of the ω phase with the α phase, as delineated in Table 1.

For the structural phase analysis of the samples, the X-ray (XRD) patterns were measured using a Siemens D-500 X-ray diffractometer (Berlin, Germany) in Cu-K_α1_ radiation. The phase analysis and calculation of the lattice parameters were carried out using the PowderCell for Windows Version 2.4.08.03.2000 program (Werner Kraus and Gert Nolze, BAM, Berlin, Germany). To determine the chemical composition of the samples, a scanning electron microscope (SEM) Tescan Vega TS5130 MM (Brno, Czech Republic) with the INCA Energy 450+ microanalysis system was used, equipped with an Oxford Instruments (Abingdon, UK) energy dispersion microanalysis prefix. The SEM FEI E-SEM XL30 instrument (manufactured by FEI, Hillsborough, OR, USA) was also used. It was equipped with an EDAX Genesis energy-dispersive X-ray (EDS) spectrometer (FEI, Hillsborough, OR, USA). Transmission electron microscopy (TEM) was made by using a TECNAI G2 FEG super TWIN (200 kV) instrument (manufactured by FEI, Hillsborough, OR, USA) equipped with the EDS spectrometer produced by EDAX (AMETEK, Inc., Berwyn, PA, USA). For the production of TEM thin foils, the twin-jet polishing was performed using a D2 electrolyte in a Struers machine (Cleveland, OH, USA). For the analysis of spot diffraction, TIA software Version 2.1 for the Tecnai microscope was applied (FEI, Hillsborough, OR, USA). Additionally, the identification of constituent phases was performed with CARINEV3 software Version 3.0 (Cyrille Boudias and Daniel Monceau Ltd., Paris, France). The decoding of the diffraction rings is carried out by the calculation of lattice spacing using ProcessDiffraction Version 8.7.1 Q software (University of California, Berkeley, CA, USA) [38].

**Figure 1:**
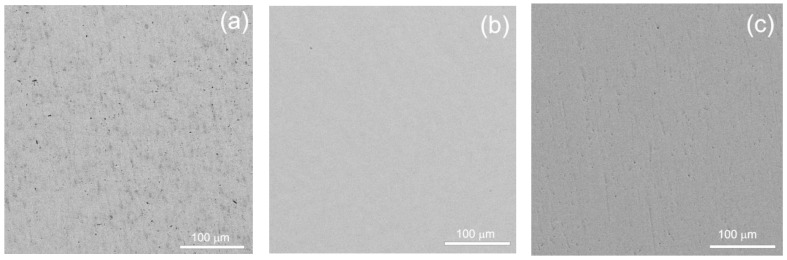
SEM micrographs of the structure of as-cast titanium alloys with (**a**) 6 wt.% Nb, (**b**) 10 wt.% Nb, and (**c**) 4 wt.% Fe in reflected electrons.

As an illustrative example, Figure 1 presents scanning electron microscopy (SEM) micrographs of the microstructure in as-cast titanium alloys with 6 wt.% and 10 wt.% niobium, as well as with 4 wt.% iron, captured in reflected electrons. Before embarking on biological investigations, it is noteworthy that the samples underwent sterilization by immersion in 70% ethanol for a duration of 3 h, followed by a meticulous drying process in a sterile atmosphere. It is worth mentioning that each test was conducted using three samples featuring the same alloy composition to ensure consistency and reliability.

### 2.2. Cytotoxicity Studies

This study closely followed the methods detailed in our prior publication [61]. In summary, red blood cells (RBCs) were isolated from C57Bl/6 mice (average weight m = 22 ± 1 g, n = 3) and suspended in a growth medium based on Dulbecco’s Modified Eagle Medium (DMEM), reaching a concentration of 6,200,000 cells per milliliter. The titanium alloys were incubated with the red blood cells for a duration of 4 h and maintained at 37 °C within an environment containing 5% carbon dioxide. For reference, cells that were incubated without alloys in the same growth medium under identical conditions served as the control group. The study’s outcome allowed for the calculation of the percentage of hemolysis.

### 2.3. Studies of Cell Adhesion and Colonization of the Surface of Titanium Alloys

The study employed C57Bl/6 mouse multipotent mesenchymal stromal cells (MMSCs) as the cellular model. A total of 20 μL of MMSCs, sourced from the N.N. Blokhin NMRC of Oncology in Moscow, Russia, were seeded onto the alloy samples. Subsequently, the samples were incubated at 37 °C in an atmosphere containing 5% carbon dioxide for a period of 30 min. The cell concentration was adjusted to 510,000 cells per milliliter. To examine the size and clustering of the cells, we utilized a LionHeart analyzer (Perkin Elmer, Shelton, CT, USA). As a control, MMSCs were seeded at the bottoms of empty wells. A portion of the alloy samples with MMSCs, as well as the control wells, was designated for the study of cell adhesion. In this phase, a 2 mL aliquot of the medium was added to the remaining wells containing cells seeded on the alloys, as well as the control wells. These samples were incubated for 14 days under the previously specified conditions to explore cell colonization. During the cell-adhesion tests, the alloy samples (and control cells seeded at the well bottom) were gently rinsed with phosphate-buffered saline (PanEco, Moscow, Russia).

To assess cell proliferation, a 2 mL volume of complete growth medium was employed, maintaining the samples at 37 °C within an environment containing 5% carbon dioxide. The medium was refreshed every 2 days to ensure optimal conditions for cell growth. The metrics for cell adhesion and colonization were quantified as a percentage of LDH activity in cells coincubated with the alloys in comparison to LDH activity in the control group (% of the control). This assessment was performed using the LDH Cytotoxicity Assay Kit from Thermo Fisher Scientific (Waltham, MA, USA), following the manufacturer’s provided instructions. For visualization, the cells were stained with Calcein AM (Sigma-Aldrich, St. Louis, MO, USA) and observed via fluorescent microscopy using the LionHeart digital microscope (Perkin Elmer, Shelton, CT, USA).

A 20 μL cell suspension was delicately applied to the surfaces of the samples and allowed to incubate for 20 min at 37 °C within an environment enriched with 5% carbon dioxide. As a positive control, a similar cell suspension was applied to the base of the plate well in the same volume, while the negative control lacked any added cells in its wells. Subsequently, 2 mL of nutrient medium were introduced into each sample and control, and the cultures were incubated for 7 days under the conditions. Following this incubation period, the old nutrient medium was carefully removed, and 500 μL of the fresh nutrient medium was replenished in both the sample and control wells to assess cell adhesion. To evaluate the colonization of the alloys’ surface by the cells, the results were analyzed 7 days after the commencement of the experiment, primarily relying on the assessment of lactate dehydrogenase (LDH) activity. An LDH analysis was performed using an analyzer in conjunction with the LionHeart digital microscope (Perkin Elmer, Shelton, CT, USA). The activity of LDH was determined within the culture medium, following artificially induced cytolysis, employing the Pierce LDH Cytotoxicity Assay Kit (Thermo Fisher Scientific, Waltham, MA, USA), adhering to the manufacturer’s specified instructions. The optical density was measured utilizing an MS Multiscan plate reader (Labsystems, Thermo Fisher Scientific, Waltham, MA, USA) and employing a 492 nm filter in comparison to a 690 nm filter within a 96-well plate. The cellular lactate dehydrogenase activity (LDH activity) was computed as the difference between the optical densities measured at distinct wavelengths.

To facilitate statistical analysis, the study calculated the mean values and their corresponding standard deviations (M ± SD) based on triplicate measurements. The results were then assessed through a comparative analysis of the LDH activity in cells after incubation with alloy samples, with the control group serving as intact cells incubated at the bottom of the wells without any alloy presence.

## 3. Results and Discussion

### 3.1. Studies of Microstructure

**Figure 2:**
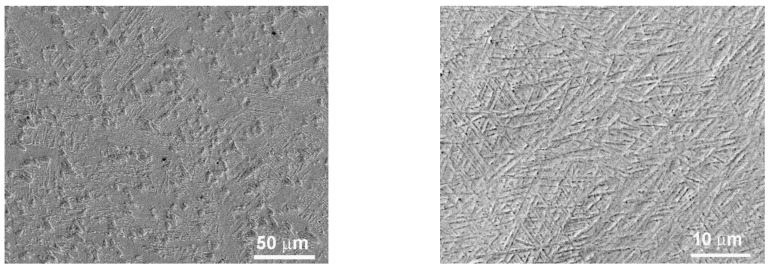
SEM micrographs of the structure of samples Ti-6% Nb (**left**) and Ti-10% Nb (**right**), annealed at 1000 °C, 24 h before HPT.

**Figure 3:**
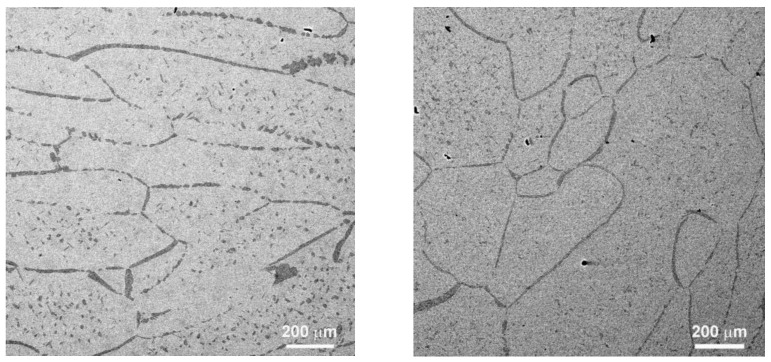
SEM micrographs of the structure of a Ti-4% Fe sample annealed at 800 °C, 100 h before HPT, at different magnifications.

**Figure 4:**
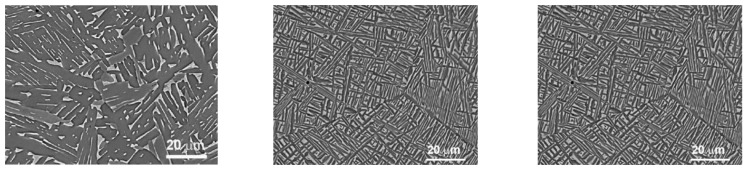
SEM micrographs of the structure of Ti-5% Nb, Ti-10% Nb, and Ti-20% Nb samples (from **left** to **right**), annealed at 800 °C, 91 h before HPT.

**Figure 5:**
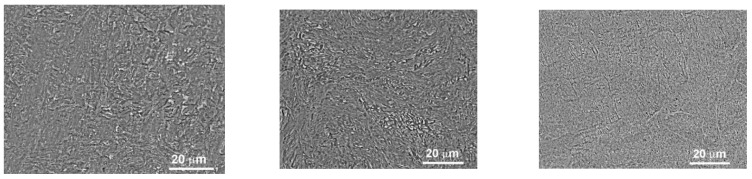
SEM micrographs of the structure of Ti-5% Nb, Ti-10% Nb, and Ti-20% Nb samples (from **left** to **right**) annealed at 800 °C, 91 h after HPT.

**Figure 6:**
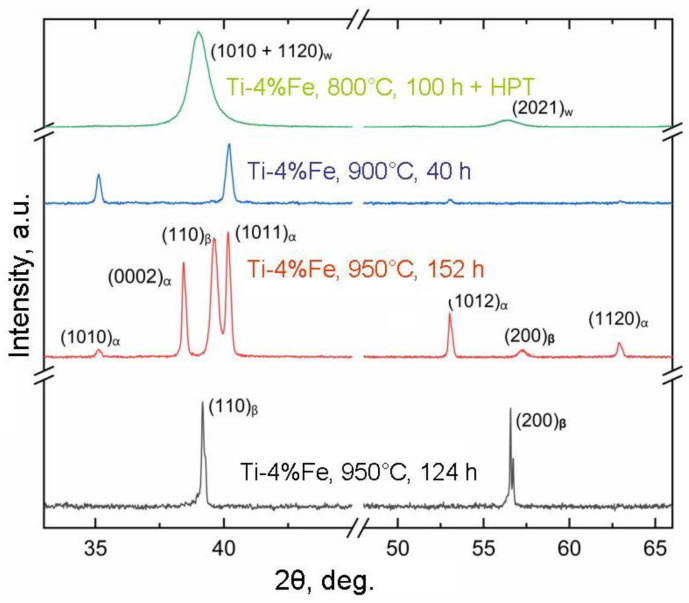
X-ray diffraction patterns for samples 1a, 2a, 3a, and 4b (for designations, see Table 1). Sample 1a is in a lower pattern. Sample 2a is the second pattern from the bottom. Sample 3a is the third pattern from the bottom. Sample 4b is the upper pattern.

**Figure 7:**
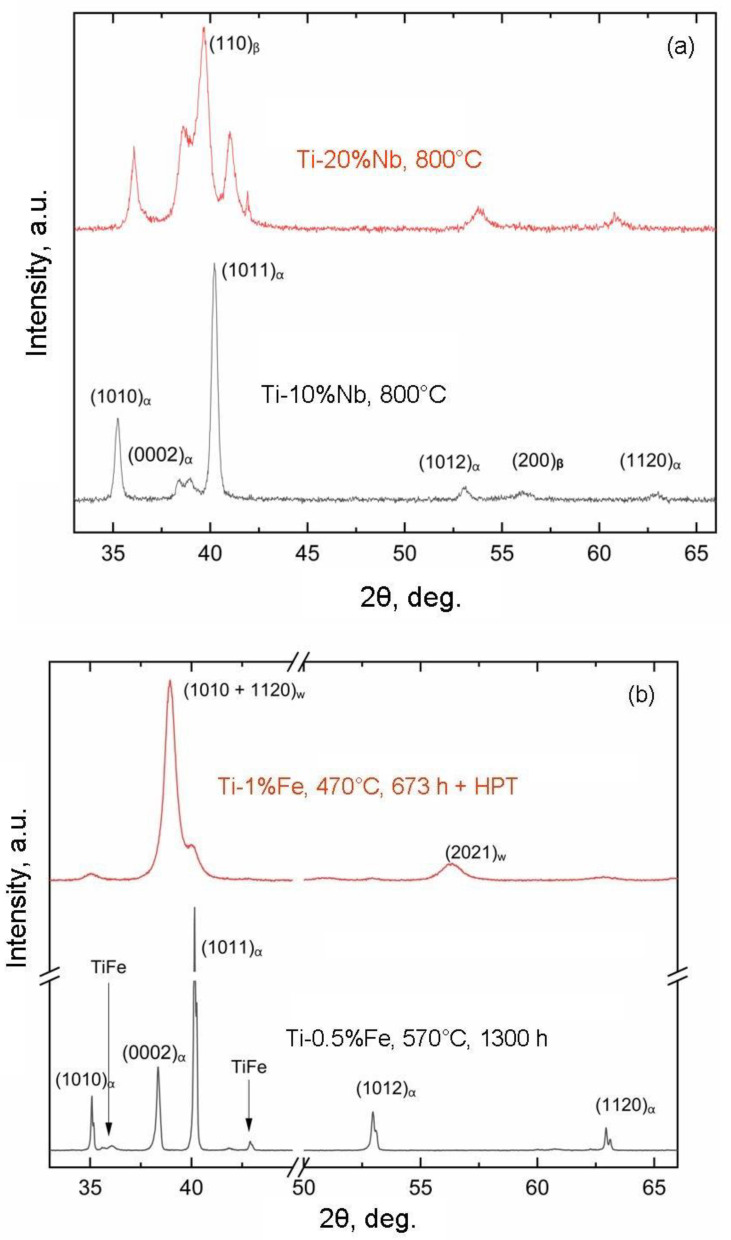
X-ray diffraction patterns for samples 41a and 41b (for designations, see Table 1). Sample 41a—**lower** pattern (**b**). Sample 41b—**upper** pattern (**a**).

**Figure 8:**
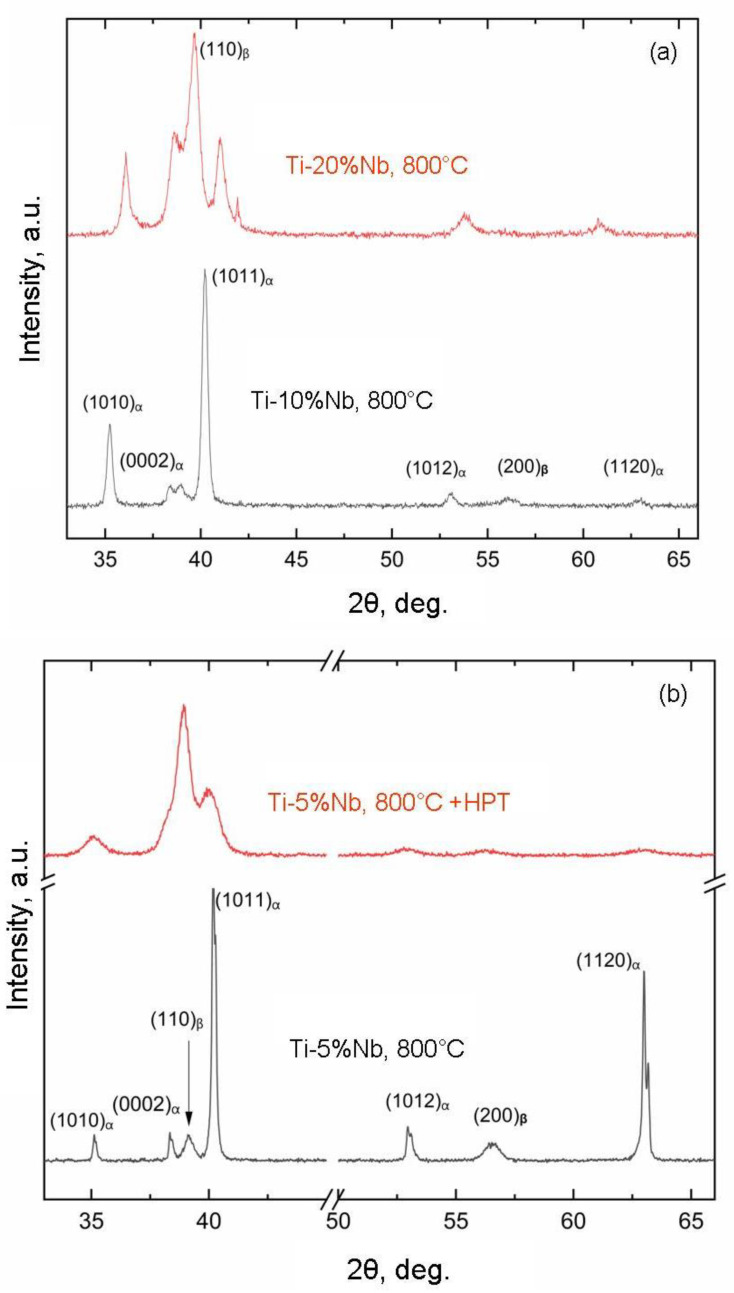
X-ray diffraction patterns for samples 64a, 65a (**a**) and 63a, 63b (**b**) (for designations, see Table 1).

**Figure 9:**
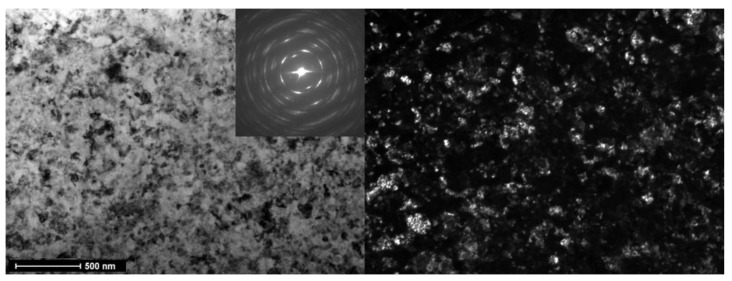
TEM Micrographs of a Ti–5 wt% Nb alloy annealed at T = 800 °C, 91 h, quenched and subjected to HPT at 7 GPa, 1 rpm, and 5 rpm (sample 63b). On the left is a bright-field image; on the right is a dark-field image of the same area. Selected area electron diffraction (SAED) is shown in the inset.

The microstructure of both annealed and HPT-treated alloys underwent comprehensive analysis, employing a combination of scanning electron microscopy (as depicted in Figure 2, Figure 3, Figure 4 and Figure 5), X-ray diffraction (shown in Figure 6, Figure 7 and Figure 8), and transmission electron microscopy, which is illustrated in Figure 9. 

In Figure 6, the X-ray diffraction patterns for samples 1a, 2a, 3a, and 4b (for specific designations, refer to Table 1) are depicted. All these samples maintain an identical iron content at 4 wt.%. However, in the case of the annealed samples (1a, 2a, 3a), there is a noticeable shift in the proportion of α and β phases. Progressing from sample 1a to sample 3a, there is a reduction in the β-phase fraction, while the α-phase fraction increases. Sample 4b, post-HPT, exhibits a composition solely comprising 100% of the ω phase. The pronounced broad peaks observed in the X-ray diffraction spectra reflect the nanosized grains constituting the ω phase within this particular sample.

Figure 7 showcases the X-ray diffraction patterns for samples 41a and 41b (see Table 1 for designations). These samples contain roughly the same iron content and were preannealed at a temperature below the eutectoid transformation temperature. In other words, after annealing, both samples feature the α phase and the TiFe intermetallic compound. These same phases are retained in the sample following HPT. However, the presence of broad peaks in the upper pattern signifies that, after HPT, both the α-phase grains and the TiFe intermetallic particles undergo significant refinement, reducing them to a nanoscale size. This implies that, in these samples, grain and particle sizes undergo variation while maintaining a consistent concentration and phase composition. Figure 8 illustrates the X-ray diffraction patterns for samples 64a, 65a (Figure 8a) and 63a, 63b (lower figure) (for specific designations, refer to Table 1). In the Ti-Nb alloy samples (63a, 64a, and 65a), the niobium concentration ranges from 5 to 20 wt.%. At the same annealing temperature, this variation results in an increase in the fraction of the β phase and a decrease in the fraction of the α phase with rising niobium content. In the Ti-Nb alloy samples (63b, 61b, 62b, and 66b), the niobium concentration ranges from 5 to 30 wt.%. Post-HPT, the ω-phase fraction increases from 0 to 100%, and, correspondingly, the α-phase fraction decreases from 100% to zero. Consequently, these samples exhibit changes in the proportion of the α and ω phases following HPT, influenced by alterations in the composition and concurrent formation of nanograins and nanoparticles.

In Figure 9, TEM micrographs of a Ti-5 wt.% Nb alloy are displayed. This alloy was initially annealed at T = 800 °C for 91 h, then swiftly quenched and subsequently subjected to HPT at 7 GPa with rotation rates of 1 rpm and 5 rpm (sample 63b). The left side presents a bright-field image, while the right side showcases a dark-field image of the same region. An inset features the selected area electron diffraction (SAED) pattern. In this sample, the α and ω phases are evenly distributed. Notably, the HPT process results in a substantial grain refinement, reducing the grain size to approximately 50 nm.

Table 2 provides data on samples that were subsequently used for biological research.

### 3.2. Studies of the Hemolysis Induced by Titanium Alloy Samples in the In Vitro Experiments

The investigation involved the assessment of optical density (OD) values for hemoglobin solutions in a physiological solution using spectrophotometry in both the control and experimental samples. Each sample, and their respective controls, was characterized by measuring OD in triplicate within the same row of the plate. Subsequently, the primary data collected were subjected to a descriptive analysis, summarizing the OD values within the wells containing experimental samples and controls (as presented in Table 3). This analysis was conducted using the “Basic Statistics/Tables” module integrated into the statistical analysis software package Statistica 6.0 (StatSoft GmbH, Hamburg, Germany).

Based on the analysis of optical density in the “intact control” and “100% hemolysis control” samples, a calibration curve was established. This curve allowed for the derivation of an equation that quantifies the relationship between the level of hemolysis and OD, achieved using specialized software package Statistica 6.0 (StatSoft GmbH, Hamburg, Germany). The complete hemolysis of the initial erythrocyte suspension was accomplished through coincubation with the detergent Triton X-100. It is well-documented that the lysis of erythrocytes induced by detergents involves the disruption of the lipid bilayer of the cell membrane. Detergents possess the capacity to interact with membrane proteins through hydrophobic bonds while simultaneously engaging with polar groups in contact with water. It is worth noting that a significant distinction exists in the effects of ionic and nonionic detergents on the erythrocyte membrane. In this study, we employed the nonionic detergent octylphenol polyethylene glycol, Triton X-100. Research has demonstrated that Triton X-100 solubilizes fewer membrane proteins compared to ionic detergents like cetyltrimethylammonium bromide. Osmotic hemolysis, induced by Triton X-100, leads to structural alterations in the band 3 protein, which constitutes the anion transport channel and is a primary integral protein, constituting roughly half of the total integral proteins in the erythrocyte membrane. Band 3 protein is highly structured, spanning the erythrocyte membrane in multiple locations and existing in dimeric and tetrameric forms. Triton X-100, when present at concentrations corresponding to the critical micelle concentration (CMC), leads to the extraction of proteins and the liquefaction of the lipid bilayer without modifying the polarity of the membrane’s hydrophobic regions. The actual breakdown of the lipid bilayer, along with the separation of lipids and proteins, commences upon the incorporation of Triton X-100 monomers into the membranes, and this interaction is characterized by a nonmicellar mechanism. In this context, the solubilization of membranes through nonionic detergents, in contrast to SDS, primarily arises through cooperative binding with the lipid components of protein-containing membranes. To achieve full hemolysis in the samples, we employed Triton X-100 at an effective concentration of 800 μmol/L. The completeness of hemolysis was verified using a Goryaev chamber, where no intact erythrocytes were observed in the “100% hemolysis control” sample after coincubation with the detergent. The resulting equation was subsequently used to calculate the level of hemolysis induced by the coincubation of mammalian erythrocytes with the studied samples. The statistical analysis results are presented in Table 4.

**Figure 10:**
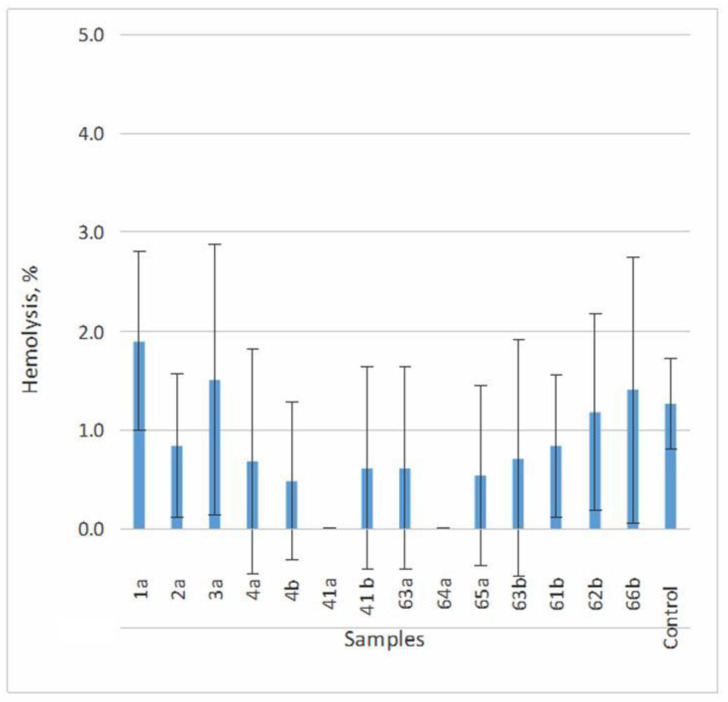
Comparative analysis of hemolysis levels induced by various alloys.

The results of the comparative analysis, as presented in Table 4 and Figure 10, clearly indicate that the average level of hemolysis induced by all the studied alloys remained consistently below 1.9%. Furthermore, a comprehensive statistical analysis revealed the absence of a significant distinction in the level of hemolysis between the wells of the spontaneous control group and those where erythrocytes were incubated with the tested compounds (*p* >> 0.05). Consequently, based on this assessment, all the alloys under investigation can be confidently categorized as biocompatible.

### 3.3. Cytotoxicity Studies

This study involved the utilization of mononuclear leukocytes obtained from the blood of healthy donors, with particular attention on the oxidative potential (OP), assessed through photometry. The outcomes of the statistical analysis, based on OD measurements conducted in triplicate for both the experimental and control samples, are presented in Table 5.

**Figure 11:**
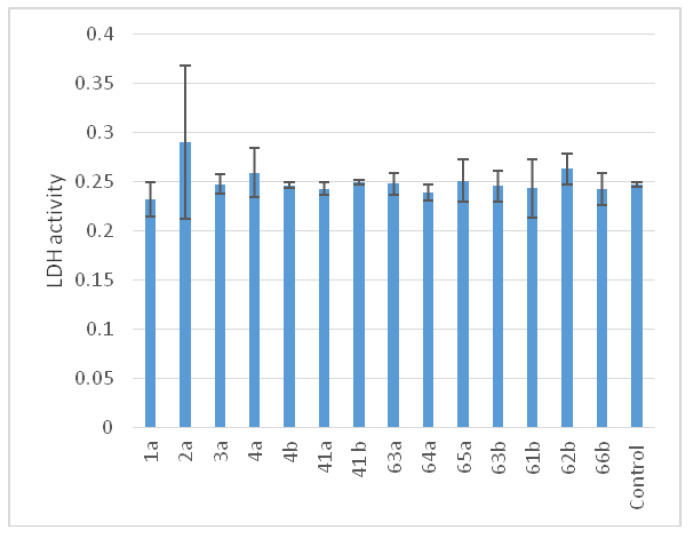
Cytotoxic activity of titanium alloy samples according to the results of a comparative analysis of the level of extracellular LDH activity in the control and after incubation with the studied samples M ± SD.

The data presented in Figure 11 and the results obtained from the comparative statistical analysis in Table 5 unequivocally reveal that the extent of extracellular LDH release in the wells where leukocytes were incubated with the investigated compounds exhibited no substantial variance compared to the control group. This compelling observation underscores the absence of any discernible membrane damage to immunocompetent human blood cells, thereby permitting the classification of all the tested samples as biocompatible in this regard.

This study delved into the assessment of the biocompatibility of titanium alloy samples, known for their enhanced physical and mechanical characteristics, by scrutinizing their impact on induced hemolysis and cytotoxicity. Through meticulous in vitro investigations employing photometry techniques with erythrocytes and mononuclear leukocytes sourced from the blood of healthy donors, the findings unequivocally indicate that none of the examined samples exhibited significant cytotoxic or hemolytic effects on mammalian cells. This compelling outcome permits the unequivocal classification of these alloys as biocompatible materials, with the acquired data suggesting their promising potential for a wide range of medical applications.

### 3.4. Study of the Influence of Titanium Alloy Surface Features on Human MHMSCs Colonization

**Figure 12:**
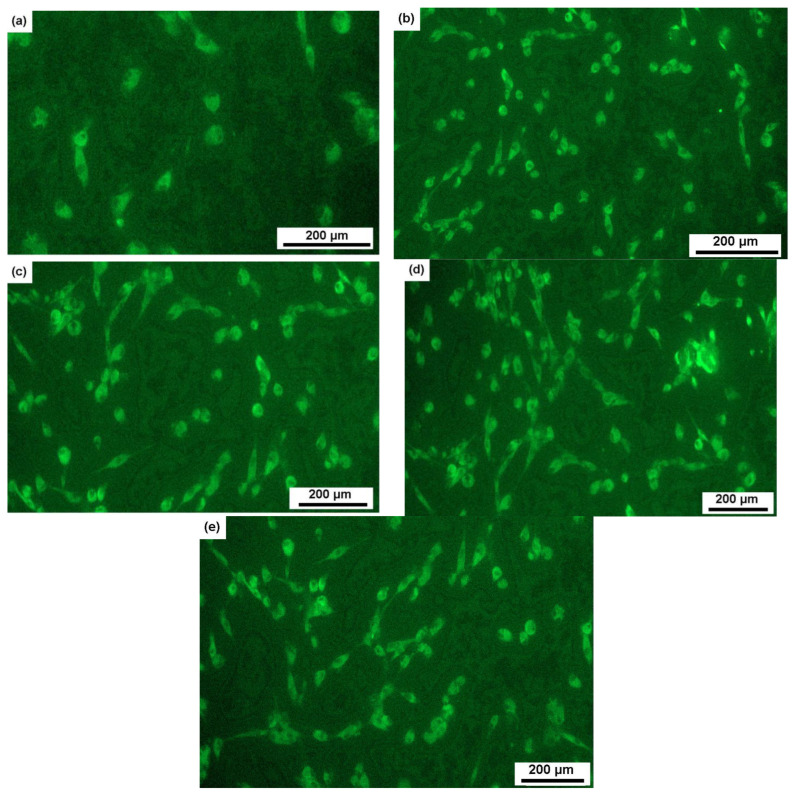
Micrographs showing the colonization of human MHMS cells on the surfaces of samples of alloys (**a**)1a (Ti-4% Fe, 950 °C, 124 h, 80% β, 20% α), (**b**) 2a (Ti-4% Fe, 950 °C, 152 h, 50% β, 50% α), (**c**) 3a (Ti-4% Fe, 900 °C, 40 h, 20% β, 80% α), and (**d**) 4b (Ti-4% Fe, 800 °C, 100 h + HPT, 100% ω) in comparison with (**e**) control.

In Figure 12, the illustration provides a visual representation of how human MHMSCs (mesenchymal stem cells) populate the surfaces of alloy samples 1a–4b when compared to the control group. It is important to note that samples 1a–4b share identical compositions, but their respective ratios of α, β, and ω phases vary, contributing to differences in cell colonization patterns.

**Figure 13:**
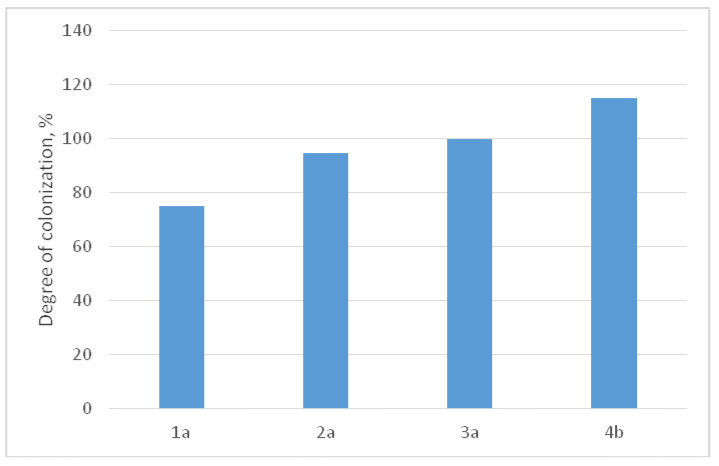
The degree of colonization of human MHMS cells on the surfaces of alloy samples 1a–4b.

The data presented in Figure 13 demonstrate a noteworthy trend: the stimulation of cell-surface colonization increases as the proportion of the α phase rises and the β phase diminishes. Notably, in a sample comprised entirely of the ω phase, the proportion of colonized surface reaches 115%, indicating the initiation of a second layer of cell growth. Figure 12 and Figure 13 offer a comparative analysis of samples with varying phase compositions. However, it is essential to highlight that, in order to induce the ω phase in these samples, a high-pressure torsion (HPT) process was employed, as the ω phase is a high-pressure one. Attaining a sufficient quantity of the ω phase through heat treatment alone is exceptionally challenging. Consequently, we are comparing samples with a coarse-grained structure (1a, 2a, and 3a) to a fine-grained sample (4b), where the grain size is approximately 50 nm.

**Figure 14:**
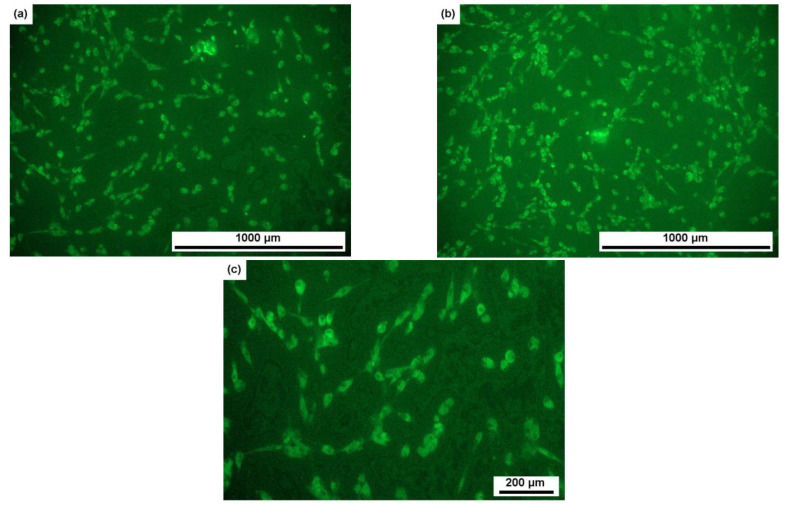
Micrographs showing colonization of human MHMS cells on the surfaces of samples of alloys (**a**) 41a (Ti-0.5% Fe, 570 °C, 1300 h, α + TiFe) and (**b**) 41b (Ti-1% Fe, 470 °C, 673 h +HPT, α + TiFe) in comparison with (**c**) control.

**Figure 15:**
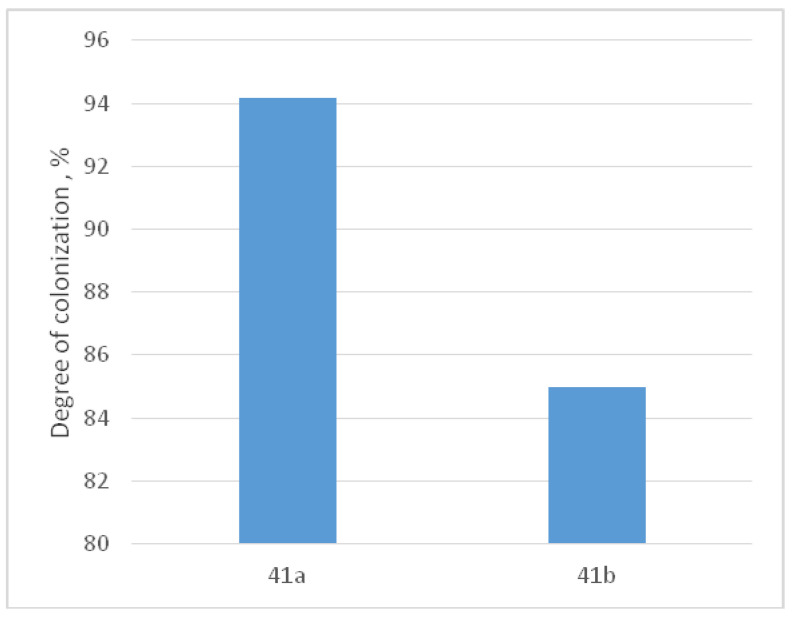
Comparative analysis of the level of LDH activity of MHMSCs on the surfaces of samples 41a and 41b, with almost the same composition, containing the same α + TiFe phases before and after HPT. Only the grain size changes (large before HPT and small ones after HPT).

In order to assess the potential impact of grain-size reduction, we conducted an experiment comparing the activity of MHMSCs on the surfaces of similarly composed samples, namely 41a (Ti-0.5 wt.% Fe) and 41b (Ti-1 wt.% Fe), both before and after HPT. These samples featured identical phase compositions, comprising primarily α titanium and a minor presence of TiFe, both before and after HPT. The sole distinction between these samples lay in their grain size, with 41a possessing larger grains following annealing and 41b featuring smaller grains after additional HPT treatment. Figure 14 showcases micrographs revealing the colonization of human MHMSCs on the surfaces of alloys 41a and 41b, compared to the control group. The data presented in Figure 14 and Figure 15 clearly indicate that the stimulation of surface colonization by human MHMSCs on samples of alloys 41a and 41b remains almost identical and even experiences a slight decrease after HPT. This suggests that the effect of reduced grain size, as depicted in Figure 14, can be disregarded, reaffirming the conclusion that the shift from a mixture of α and β phases to a 100% ω phase indeed enhances surface colonization.

**Figure 16:**
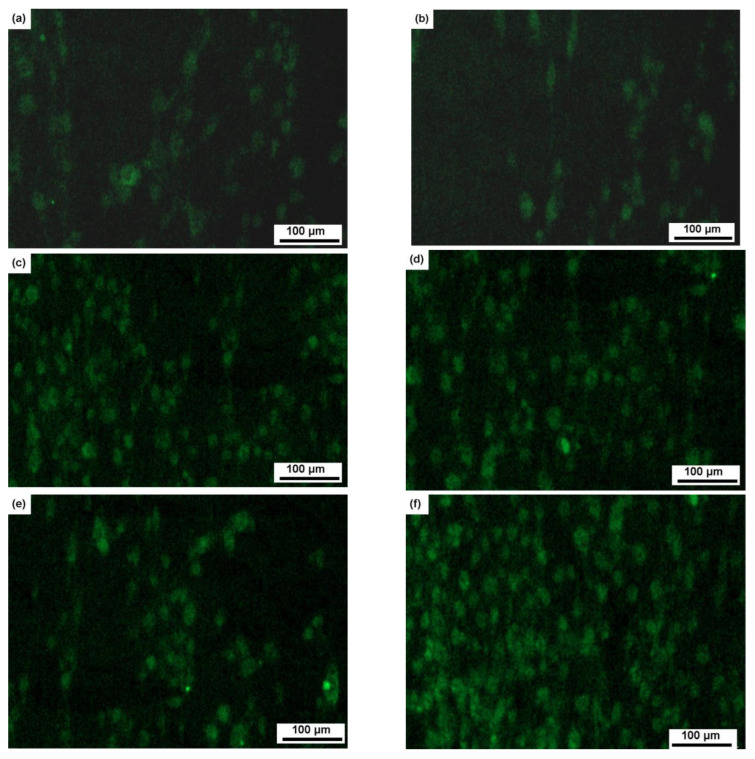

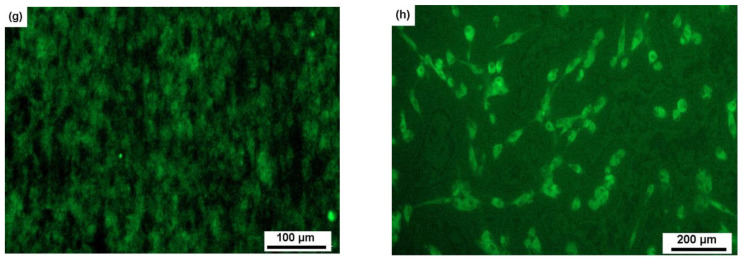
Colonization of human MHMSCs on the surfaces of samples (**a**) 63a (Ti-5% Nb, 800 °C, 91 h, α + β, 20% β, 80% α), (**b**) 64a (Ti-10% Nb, 800 °C, 91 h, α + β, 50% β, 50% α), (**c**) 65a (Ti-20% Nb, 800 °C, 91 h, α + β, 80% β, 20% α), (**d**) 63b (Ti-5% Nb, 800 °C, 91 h + HPT, 50% α + 50% ω), (**e**) 61b (Ti-6% Nb, 1000 °C, 24 h + HPT, α + α’, no ω), (**f**) 62b (Ti-10% Nb, 1000 °C, 24 h + HPT, 10% α + 90% ω), and (**g**) 66b (Ti-30% Nb, 600 °C, 768 h + HPT, 100% ω) before and after HPT in the titanium alloys containing niobium at different concentrations, with differences in the proportion of phases and grain size in comparison with (**h**) the control.

**Figure 17:**
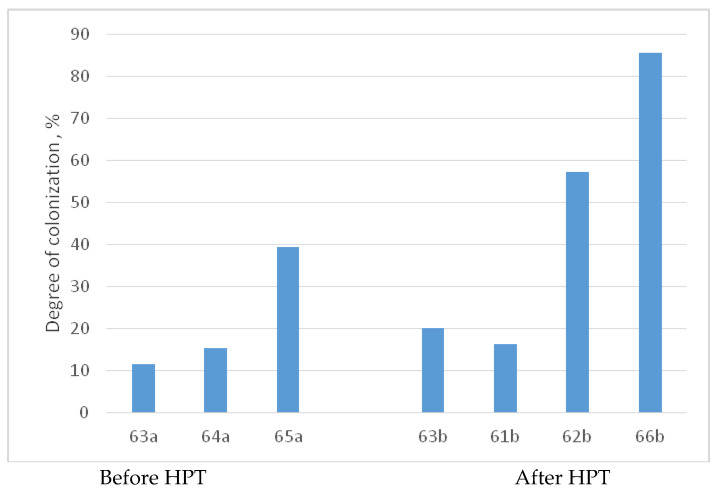
Comparative analysis of the level of LDH activity of MHMSCs on the surfaces of samples 63a–66b before and after HPT.

Figure 16 offers micrographs depicting the colonization of human MHMS cells on the surfaces of titanium–niobium alloy samples. Before undergoing HPT, in the series comprising 63a, 64a, and 65a, an increase in niobium concentration results in a corresponding increase in the proportion of the β phase and a decrease in the proportion of the α phase. Post HPT, the series 63b, 61b, 62b, and 66b, with a higher niobium concentration, exhibit an augmented presence of the ω phase and a reduced proportion of the α phase. Notably, in both scenarios, the level of colonization experiences a notable uptick with increasing niobium concentration. Figure 17 provides a comparative analysis of LDH activity levels in MHMSCs on the surfaces of samples 63a–66b, both before and after HPT treatment.

Figure 17 reveals significant trends in the impact of niobium concentration on alloy characteristics. Before HPT, the data shows a clear pattern: as niobium concentration increases, the proportion of the β phase rises, while the proportion of the α phase decreases. After HPT treatment, a similar pattern emerges but with a twist: a higher niobium concentration leads to an increased presence of the ω phase, while the α phase diminishes. Notably, in both scenarios, greater niobium concentration corresponds to enhanced colonization. In simpler terms, as the proportion of the ω phase increases, colonization improves, whether in alloys containing iron or niobium.

## 4. Conclusions

The adhesion of host cells to implant surfaces, and their subsequent colonization, plays a pivotal role in determining the functionality of metal implants postreconstructive surgery. Among the key materials employed for crafting these implants are titanium alloys, which have earned their place in the spotlight. Titanium possesses three crystallographic modifications with distinct lattices—namely the α, β, and ω phases. In this study, we embarked on a pioneering investigation into how the volume fractions of these phases in binary titanium alloys influence cell adhesion to implant surfaces. To manipulate the volume fractions of the α, β, and ω phases, we implemented alterations in chemical composition and customized thermal and mechanical treatment protocols. Notably, we observed, based on the measured values of induced hemolysis and cytotoxicity, that all the binary alloys under scrutiny fall into the category of biocompatible materials. Consequently, our findings unveiled a compelling correlation between surface colonization by human multipotent mesenchymal stromal cells and the volume fraction of the ω phase. As this fraction increased, and simultaneously as the α and β phases decreased, a surge in colonization was observed. It is worth mentioning that the high-pressure ω phase, although metastable after pressure release, lingers in binary Ti alloys until subjected to temperatures ranging from 300–600 °C [62]. This research allows us to disentangle the pure influence of the α-, β-, and ω-phase portions, thus eliminating other variables like composition and grain size from the equation. The outcomes regarding the contributions of various Ti phases to biocompatibility equip materials scientists, engineers, and medical practitioners with valuable insights to develop enhanced Ti-based alloys for medical applications. Furthermore, the auspicious properties of the ω phase open up exciting possibilities for the emergence of a third generation of biocompatible Ti alloys, ushering in the era of ω-based materials after the initial α-Ti alloys of the first generation and β alloys of the second.

## Figures and Tables

**Table 1:** Compositions and conditions of the thermal and mechanical treatment of samples that were subsequently used for biomedical studies.

Sample Denomination	Composition, wt.%	Conditions of Annealing and Postprocessing
1a	Ti-4% Fe	950 °C, 124 h
2a	Ti-4% Fe	950 °C, 152 h
3a	Ti-4% Fe	900 °C, 40 h
4b	Ti-4% Fe	800 °C, 100 h + HPT
41a	Ti-0.5% Fe	570 °C, 1300 h
41b	Ti-1% Fe	470 °C, 673 h + HPT
63a	Ti-5% Nb	800 °C, 91 h
64a	Ti-10% Nb	800 °C, 91 h
65a	Ti-20% Nb	800 °C, 91 h
63b	Ti-5% Nb	800 °C, 91 h + HPT
61b	Ti-6% Nb	1000 °C, 24 h + HPT
62b	Ti-10% Nb	1000 °C, 24 h + HPT
66b	Ti-30% Nb	600 °C, 768 h + HPT

**Table 2:** Phase composition of samples that were subsequently used for biological studies.

Sample Denomination	Composition, wt.%	Conditions of Annealing and Postprocessing	Phases in the Sample
1a	Ti-4% Fe	950 °C, 124 h	α + β, 80% β, 20% α
2a	Ti-4% Fe	950 °C, 152 h	α + β, 50% β, 50% α
3a	Ti-4% Fe	900 °C, 40 h	α + β, 20% β, 80% α
4b	Ti-4% Fe	800 °C, 100 h + HPT	100% ω phase
41a	Ti-0.5% Fe	570 °C, 1300 h	α phase v + TiFe, large grains
41b	Ti-1% Fe	470 °C, 673 h + HPT	α phase + TiFe, fine grains
63a	Ti-5% Nb	800 °C, 91 h	α + β, 20% β, 80% α
64a	Ti-10% Nb	800 °C, 91 h	α + β, 50% β, 50% α
65a	Ti-20% Nb	800 °C, 91 h	α + β, 80% β, 20% α
63b	Ti-5% Nb	800 °C, 91 h + HPT	50% α + 50% ω
61b	Ti-6% Nb	1000 °C, 24 h + HPT	α + α’, no ω
62b	Ti-10% Nb	1000 °C, 24 h + HPT	10% α + 90% ω
66b	Ti-30% Nb	600 °C, 768 h + HPT	100% ω

**Table 3:** Optical density in wells in alloy samples and in the control.

Sample	Optical Density					*p*
	1	2	3	M	SD	
1a	0.093	0.126	0.12	0.113	0.017578	0.896
2a	0.12	0.261	0.132	0.171	0.078173	0.941
3a	0.13	0.138	0.118	0.128667	0.010066	0.918
4a	0.168	0.135	0.118	0.140333	0.025423	0.931
4b	0.128	0.124	0.13	0.127333	0.003055	0.917
41a	0.131	0.119	0.122	0.124	0.006245	0.912
41 b	0.131	0.132	0.128	0.130333	0.002082	0.921
63a	0.119	0.128	0.141	0.129333	0.01106	0.919
64a	0.129	0.114	0.116	0.119667	0.008145	0.906
65a	0.156	0.115	0.125	0.132	0.021378	0.921
63b	0.11	0.142	0.128	0.126667	0.016042	0.915
61b	0.159	0.109	0.106	0.124667	0.029771	0.910
62b	0.128	0.145	0.159	0.144	0.015524	0.937
66b	0.115	0.114	0.142	0.123667	0.015885	0.911
Spontaneous control	0.127	0.126	0.131	0.128	0.002646	-
Intact control (0% hemolysis)	0.092	0.097	0.091	0.093333	0.003215	-
Control with Triton-X (100% hemolysis)	2.145	2.221	2.174	2.18	0.038354	-

**Table 4:** Statistical analysis of the results of studying the level of hemolysis induced by samples of alloys with improved physical and mechanical characteristics in vitro.

Sample	M	SD	*p* vs. Control
1a	1.9	0.9	0.110
2a	0.8	0.7	0.380
3a	1.5	1.4	0.070
4a	0.7	1.1	0.384
4b	0.5	0.8	0.363
41a	0.0	0.0	0.156
41 b	0.6	1.0	0.185
63a	0.6	1.0	0.185
64a	0.0	0.0	0.155
65a	0.5	0.9	0.211
63b	0.7	1.2	0.370
61b	0.8	0.7	0.529
62b	1.2	1.0	0.486
66b	1.4	1.3	0.432
Control	1.3	0.5	-

**Table 5:** Results of the statistical analysis of extracellular LDH activity triplets data after incubation with titanium alloy samples and in an intact control.

Sample	M	SD	*p* vs. Control
1a	0.232	0.018	0.997
2a	0.290	0.078	0.963
3a	0.248	0.010	0.999
4a	0.259	0.025	0.996
4b	0.246	0.003	1.000
41a	0.243	0.006	1.000
41b	0.249	0.002	1.000
63a	0.248	0.011	1.000
64a	0.239	0.008	0.999
65a	0.251	0.021	0.998
63b	0.246	0.016	0.999
61b	0.244	0.030	0.996
62b	0.263	0.016	0.998
66b	0.243	0.016	0.999
Control	0.247	0.003	-

## Data Availability

Data are contained within the article.
